# Systematic Review of Sub-microscopic *P. vivax* Infections: Prevalence and Determining Factors

**DOI:** 10.1371/journal.pntd.0003413

**Published:** 2015-01-08

**Authors:** Qin Cheng, Jane Cunningham, Michelle L. Gatton

**Affiliations:** 1 Drug Resistance and Diagnostics, Australian Army Malaria Institute, Enoggera, Brisbane, Australia; 2 QIMR Berghofer Medical Research Institute, Brisbane, Australia; 3 Global Malaria Program, World Health Organization, Geneva, Switzerland; 4 School of Public Health and Social Work, Queensland University of Technology, Brisbane, Australia; Federal University of Sao Paulo, Brazil

## Abstract

**Background:**

Sub-microscopic (SM) Plasmodium infections represent transmission reservoirs that could jeopardise malaria elimination goals. A better understanding of the epidemiology of these infections and factors contributing to their occurrence will inform effective elimination strategies. While the epidemiology of SM *P. falciparum* infections has been documented, that of SM *P. vivax* infections has not been summarised. The objective of this study is to address this deficiency.

**Methodology/Principal Findings:**

A systematic search of PubMed was conducted, and results of both light microscopy (LM) and polymerase chain reaction (PCR)-based diagnostic tests for *P. vivax* from 44 cross-sectional surveys or screening studies of clinical malaria suspects were analysed. Analysis revealed that SM *P. vivax* is prevalent across different geographic areas with varying transmission intensities. On average, the prevalence of SM *P. vivax* in cross-sectional surveys was 10.9%, constituting 67.0% of all *P. vivax* infections detected by PCR. The relative proportion of SM *P. vivax* is significantly higher than that of the sympatric *P. falciparum* in these settings. A positive relationship exists between PCR and LM *P. vivax* prevalence, while there is a negative relationship between the proportion of SM *P. vivax* and the LM prevalence for *P. vivax*. Amongst clinical malaria suspects, however, SM *P. vivax* was not identified.

**Conclusions/Significance:**

SM *P. vivax* is prevalent across different geographic areas, particularly areas with relatively low transmission intensity. Diagnostic tools with sensitivity greater than that of LM are required for detecting these infection reservoirs. In contrast, SM *P. vivax* is not prevalent in clinical malaria suspects, supporting the recommended use of quality LM and rapid diagnostic tests in clinical case management. These findings enable malaria control and elimination programs to estimate the prevalence and proportion of SM *P. vivax* infections in their settings, and develop appropriate elimination strategies to tackle SM *P. vivax* to interrupt transmission.

## Author Summary

Light microscopy (LM) has been the mainstay of malaria diagnosis for case management and surveillance. The introduction of molecular based diagnostics such as polymerase chain reaction (PCR) in the 1990s resulted in increased reporting of Plasmodium infections in communities compared to LM, indicating that sub-microscopic (SM) Plasmodium infections are endemic in a variety of different settings. The prevalence and transmission potential of SM *P. falciparum* was reviewed; however, information on the prevalence and distribution of SM *P. vivax* in different settings is not available. In this article, the authors analysed LM and PCR results described in 38 publications (44 studies), and revealed that SM *P. vivax* is highly prevalent across different geographic areas with varying transmission intensities. On average, these infections made up 67% of *P. vivax* infections detected by PCR in cross-sectional surveys, and were relatively more prevalent in areas where malaria was under control and moving towards elimination. By contrast, SM *P. vivax* is not prevalent in clinical malaria suspects. These findings highlight that for detection of clinical malaria LM and rapid diagnostic tests (RDTs) are adequate, however, to detect very low density infections that are largely asymptomatic but still contribute to transmission, more sensitive diagnostic tools are needed.

## Introduction

The global malaria incidence and death rates have decreased in recent years [1] due to increasing funding and political commitment, as well as implementation of artemisinin combination therapy, better access to diagnostics and vector control interventions such as insecticide treated bed nets and indoor residual spray. As a result, many countries/regions are planning or have already committed to eliminating malaria. In these areas the malaria surveillance programs that generate information on malaria cases, burden and transmission trends need to be strengthened and extended to include case and foci investigations [2]. These focused investigations play a pivotal role in informing malaria elimination action plans and directing resources.

The effectiveness of malaria surveillance depends on the performance of surveillance tools. Light microscopy (LM) has been the main surveillance tool over the past decades. LM provides important epidemiological information such as malaria incidence rates, burden and relative species composition. However, since the introduction of molecular based diagnostics, e.g. polymerase chain reaction (PCR) in the 1990s, there has been increased reporting of malaria infections in communities which are detected by PCR, but not by LM. These reports demonstrate LM's limitation in detecting infections with low parasite densities; levels well below the threshold for symptomatic malaria, but of sufficient density to enable transmission of parasites to mosquitoes [3], [4]. Therefore, it is important to understand the epidemiology of these sub-microscopic (SM) malaria infections in different settings and their role in maintaining malaria transmission, particularly in the context of elimination strategies.

SM *P. falciparum* infections are well documented. A systematic review and meta-analysis of *P. falciparum* LM and PCR prevalence data revealed that on average, the prevalence of LM was only 50.8% of that measured by PCR [5]. This suggests that half of all detected *P. falciparum* infections were SM. The meta-analysis revealed that the SM *P. falciparum* is more common in adults, in areas with low transmission intensities and in chronic infections [6]. Based on findings of earlier experimental studies, Okell and colleagues estimated that SM *P. falciparum* contributes between 20% and 50% of human to mosquito transmission in areas with low and very low transmission intensity [6]. This has great implications for malaria control and elimination programs because SM *P. falciparum* infections cannot be readily detected by diagnostic tools commonly used for case management or field surveillance (such as LM and rapid diagnostic tests, RDTs) and these undetected large reservoirs of SM *P. falciparum* can maintain low level of transmission and seed outbreaks [7].

In contrast to *P. falciparum*, the prevalence and distribution of SM *P. vivax* has not been systematically analysed despite increasing reports of asymptomatic and SM *P. vivax*. This is a major gap in moving forward with malaria elimination in certain regions since it is likely that the epidemiology of SM *P. vivax* is different to that of *P. falciparum* due to several unique biological features of *P. vivax*. For example, parasite invasion of Duffy positive reticulocytes is thought to contribute to the observed overall lower parasite densities in *P. vivax* infections compared to *P. falciparum* infections, thus heightening the theoretical likelihood of having SM infections. Other features such as relapses may affect the speed at which host immunity develops, which in turn may affect parasite density. Mature gametocytes are present earlier in *P. vivax* infections meaning they can infect mosquitoes at an early stage of infection, with SM *P. vivax* gametocytes shown to be able to successfully infect mosquitoes [8], [9]. Dormant *P. vivax* parasite stages in the liver (hypnozoites) that can activate at variable periodicity depending on geographical region [10], means that untreated SM infections will continue to relapse in the future with the potential to continue transmission during each relapse. Therefore, SM *P. vivax* poses a major challenge to malaria control and elimination programs in areas where *P. vivax* is endemic.

In this study we reviewed published literature and analysed the prevalence and relative proportion of SM *P. vivax* infections across different transmission settings. We also investigated factors associated with the occurrence of SM *P. vivax* infections. These findings can support the reorientation of malaria control programmes towards elimination of *P. vivax*.

## Methods

### Literature search

A literature search was conducted in PubMed using the search terms “vivax, PCR, survey” in Jan 2014. These initial publications (excluding 3 non-English papers) were then carefully reviewed and selected according to the following inclusion criteria: 1) Data were collected either from cross-sectional surveys of a population or a representative subset of population at one point in time or from screening studies of clinical malaria suspects, 2) Data include separate microscopy and PCR-based results for *P. vivax* in the same setting (RDT results were not considered), and 3) At least one *P. vivax* infection was detected by either PCR or LM.

### Data analysis

When results of multiple surveys were reported in a single paper, either in different locations or in different season, data from these surveys were combined if the authors did not report a significant difference in prevalence between different locations or seasons. However, if prevalence was reported to be significantly different between locations or seasons by the authors each survey was included separately, unless 1) the number of samples in each location/season was <50 and 2) there were no data provided for each individual location/season.

### Terminology

1. Microscopy method refers to detection of *Plasmodium spp*. using light microscopy (LM) examination of thick and thin blood smears following the WHO recommended protocol.
2. PCR method refers to detection of *Plasmodium spp*. by amplification of any parasite gene using any PCR-based technology, including conventional single round, multiplex, nested, semi-nested PCR, real time quantitative PCR and ligase detection reaction-fluorescent microsphere assay (LDR-FMA).
3. Parasite prevalence determined by LM and PCR. This was calculated as: total number of positive samples/total number of samples examined ×100%.
4. Prevalence of sub-microscopic (SM) infections. The SM prevalence was calculated as: PCR prevalence – LM prevalence. In surveys where LM prevalence was higher than PCR prevalence, the prevalence of SM is considered as 0.
5. Relative proportion of sub-microscopic (SM) infections. This was calculated as: SM prevalence/PCR prevalence ×100%.

#### Statistical tests

Paired comparisons of LM and PCR, and *P. falciparum* and *P. vivax* SM prevalence or SM proportion for all sites were tested using Wilcoxon matched pairs signed rank test (GraphPad Prism). Comparisons of LM, PCR and SM prevalence or relative SM proportions between different age groups and laboratory methods were performed using the Mann Whitney test.

#### Regression analysis

The relationship between LM and PCR prevalence was assessed using linear regression of the log_10_ transformed values. Log transformed values were used to resolve heteroscedasticity. The relationship between LM prevalence and proportion of SM infections was analysed using a generalized linear model with a gamma distribution and log link function.

## Results

### Search outcome and data grouping

The PubMed search produced a list of 139 publications, of which 38 met the inclusion criteria. Twenty five publications [11], [12], [13], [14], [15], [16], [17], [18], [19], [20], [21], [22], [23], [24], [25], [26], [27], [28], [29], [30], [31], [32], [33], [34], [35] reported findings of 31 cross-sectional surveys of different populations (including 29 cross-sectional surveys, one reactive case investigation and one cohort study) that were conducted by household or village based or random sampling. The remaining 13 publications [18], [36], [37], [38], [39], [40], [41], [42], [43], [44], [45], [46], [47] reported findings of fever/or clinically suspected malaria patient screening. LM and PCR results reported in these two groups of publications were analysed separately. The location, year and references of these studies are summarised in Table 1.

**Table 1:** Summary of location, year and references of the selected surveys.

Region	Country - reference No	Area	Year of survey	Reference
**Cross-sectional surveys**				
Africa	Angola-11	Caxito, Mabubas, Ucua	2010	[11]
ASIA	Bangladesh-12a	Bandarban district (4 sub-districts)	2007–2008	[12]
ASIA	Bangladesh-12b	Bandarban district (3 sub-districts)	2007	[12]
ASIA	Cambodia-13	Rattanakiri	2001	[13]
ASIA	Cambodia-14a	Rattanakiri	2001	[14]
ASIA	Cambodia-14b	Rattanakiri	2001	[14]
ASIA	Indonesia-15	Flories Island	2008	[15]
ASIA	Indonesia-16	Aceh	2010	[16]
ASIA	Lao-17	Xepon district	2006	[17]
ASIA	Malaysia-18	Sabah	NR	[18]
ASIA	Thailand-19	Kanchanaburi, Pathumthani, Nakornpathom	2009	[19]
South America	Brazil-20a	Ji-Parana	2000	[20]
South America	Brazil-20b	Portuchuelo	1998	[20]
South America	Brazil-20c	Portuchuelo	1999	[20]
South America	Brazil-21	Para State	NR	[21]
South America	Brazil-22	Amazon state	NR	[22]
South America	Brazil-23a	Riverine-ABD	2002–2003	[23]
South America	Brazil-23b	Riverine-CE	2002–2004	[23]
South America	Brazil-24	Porto Velho, Rondonia	2004–2007	[24]
South America	Brazil-25	Espirito Santo	2001	[25]
South America	Brazil-26	Amazonas State	2008	[26]
South America	Peru-27	Iquitos	1999	[27]
South America	Peru-28	Bellavista	2010	[28]
South America	Venezuela-29*	Amazonas and Sucre state	NR	[29]
South Pacific	PNG-30	East Sepik	1996	[30]
South Pacific	PNG-31	North of Madang	2000	[31]
South Pacific	PNG-32a	Central Sepik, Sepik River	2005	[32]
South Pacific	PNG-32b	Central Sepik, Foot hills	2005	[32]
South Pacific	PNG-33	Maprik District	2006	[33]
South Pacific	PNG-34	Wosera	2001–2003	[34]
South Pacific	Solomon Is-35**	Temotu	2008	[35]
**Screening clinical malaria suspects**				
Africa	Mauritania-36	Nouakchott	2007	[36]
Africa	Mauritania-37	Nouakchott	2009	[37]
Africa	Sudan-38	Refugee camps	1997	[38]
Asia	India-39	Didori and Shivpuri	2009	[39]
Asia	Malaysia-18	Hospitals	NR	[18]
Asia	Myanmar-40	Yangon and Mandalay	2000	[40]
Asia	Pakistan-41	Khyber Pakhtunkhwa, Sindh, Balochistan, Punjab, Islamabad	2011	[41]
Asia	Thailand-42	Northern Thailand	NR	[42]
Asia	Thailand-43	Umpang	2010	[43]
Europe	Italy-44	Parma	2000–2007	[44]
Europe	Italy-45	Parma	2005–2006	[45]
Europe	Spain-46	Hospitals	1997–1998	[46]
South America	Brazil-47	Amazone	2006–2007	[47]

NR: not reported

*223/295 samples were randomly selected

**PCR rates are estimates based on PCR results of a random sample set.

### Prevalence of *P. vivax* and *P. falciparum* infections in communities

**Figure 1:**
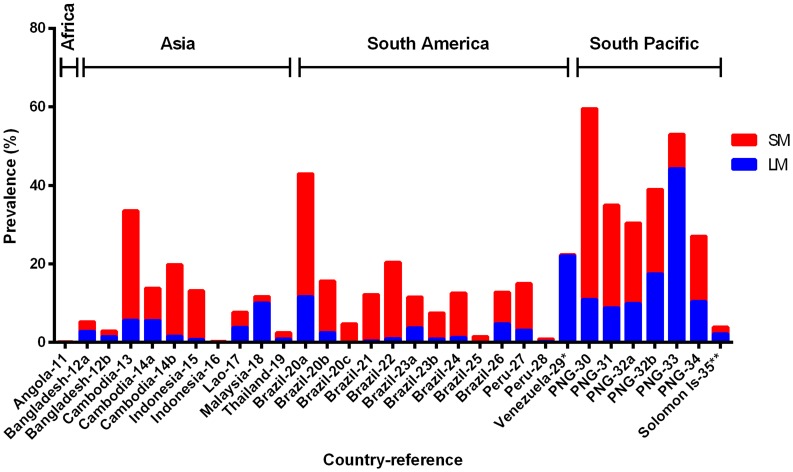
Prevalence of LM (light microscopy, blue bar) and SM (sub-microscopy, red bar) *P. vivax* in cross-sectional surveys. The total height of each bar (blue + red) represents the PCR prevalence. Countries where data were collected and their corresponding references (detailed in Table 1) are shown on the x-axis.

The 31 cross-sectional community surveys were conducted in 12 countries (Table 1). Twenty eight of these surveys were conducted between 1996 and 2010. Survey year was not described for the remaining three surveys (Table 1). The number of samples tested by PCR in each survey ranged from 98 to 3316 (median = 337, interquartile range: 252 to 1269). The LM prevalence of *P. vivax* ranged from 0.0% to 44.3% in geographically different settings, while the corresponding PCR prevalence for *P. vivax* in these same settings ranged from 0.2% to 59.5%. Overall across all sites, the PCR prevalence of *P. vivax* was significantly higher than that of LM (Wilcoxon matched-pairs signed rank test, P<0.0001, Fig. 1), i.e. PCR detected a significantly higher number of *P. vivax* infections than LM.

**Figure 2:**
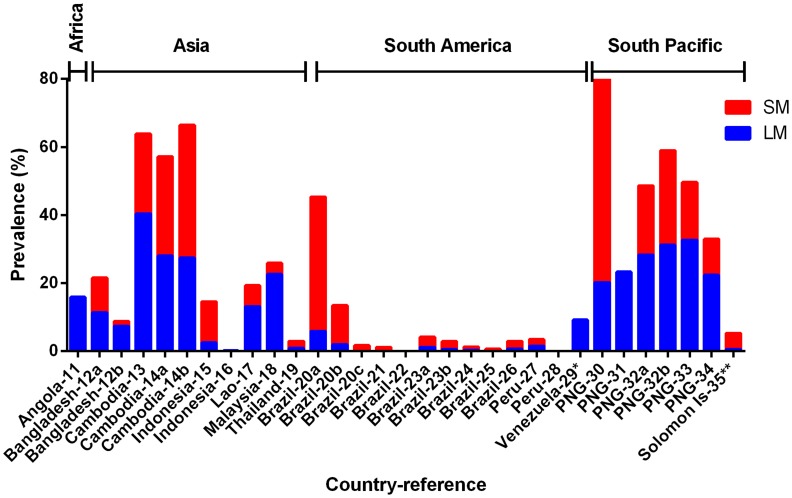
Prevalence of LM (light microscopy, blue bar) and SM (sub-microscopy, red bar) of sympatric *P. falciparum* in cross-sectional surveys. Total height of each bar (blue + red) represents the PCR prevalence. Countries where data were collected and their corresponding references (detailed in Table 1) are shown on the x-axis.

The prevalence of sympatric *P. falciparum,* including *P. falciparum* in mixed species infections, was also analysed for 30 of the 31 surveys as a comparison. Prevalence of *P. falciparum* determined by LM ranged from 0.0 to 40.4% while PCR prevalence of *P. falciparum* PCR ranged from 0.0 to 81.5%. Similar to *P. vivax*, the overall prevalence of *P. falciparum* determined by PCR was significantly higher than that of LM (Wilcoxon matched-pairs signed rank test, P<0.0001, Fig. 2).

### Prevalence and relative proportion of SM *P. vivax* and *P. falciparum* infections

**Figure 3:**
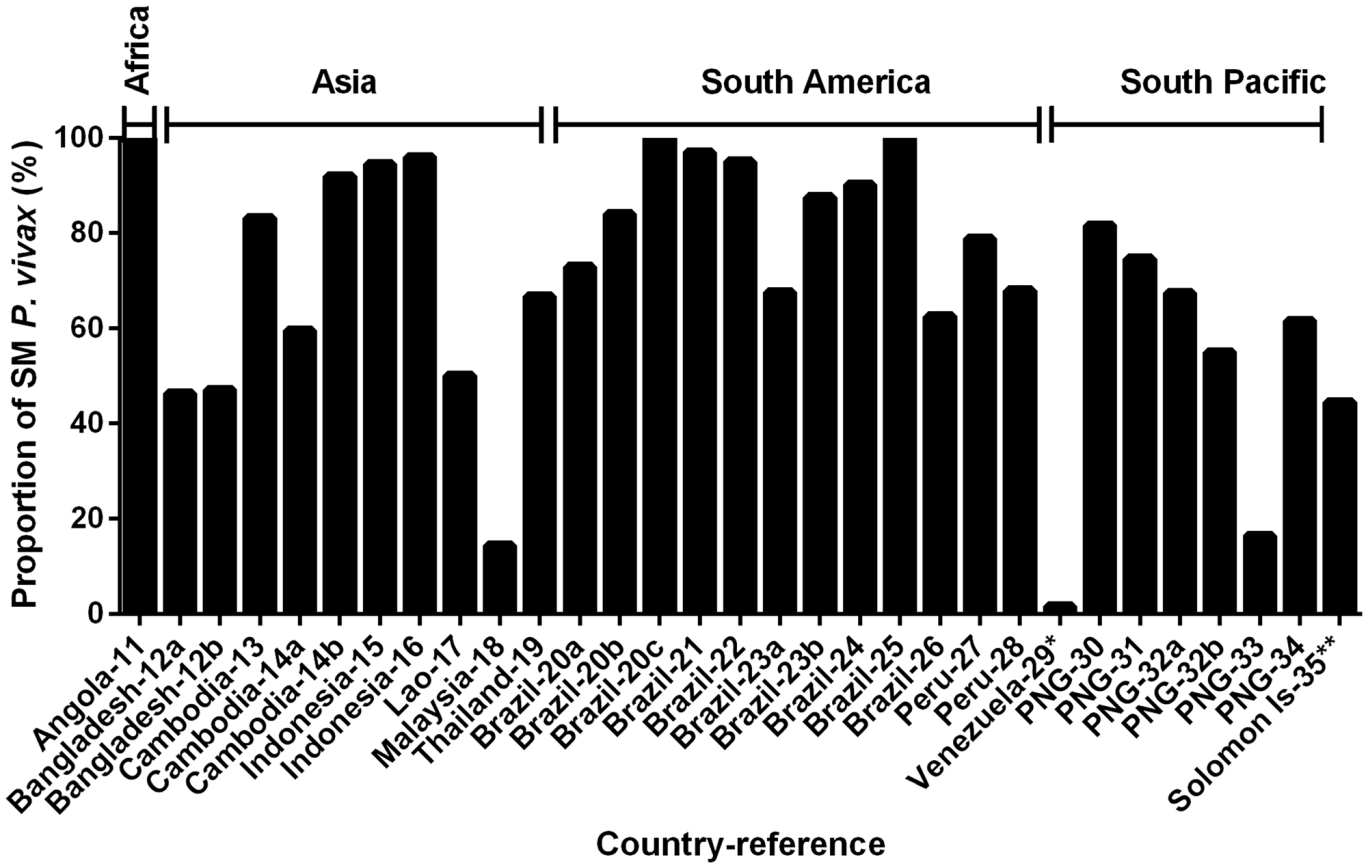
Relative proportion of SM (sub-microscopy) *P. vivax* in cross-sectional surveys. Countries where data were collected and their corresponding references (detailed in Table 1) are shown on the x-axis.

**Figure 4:**
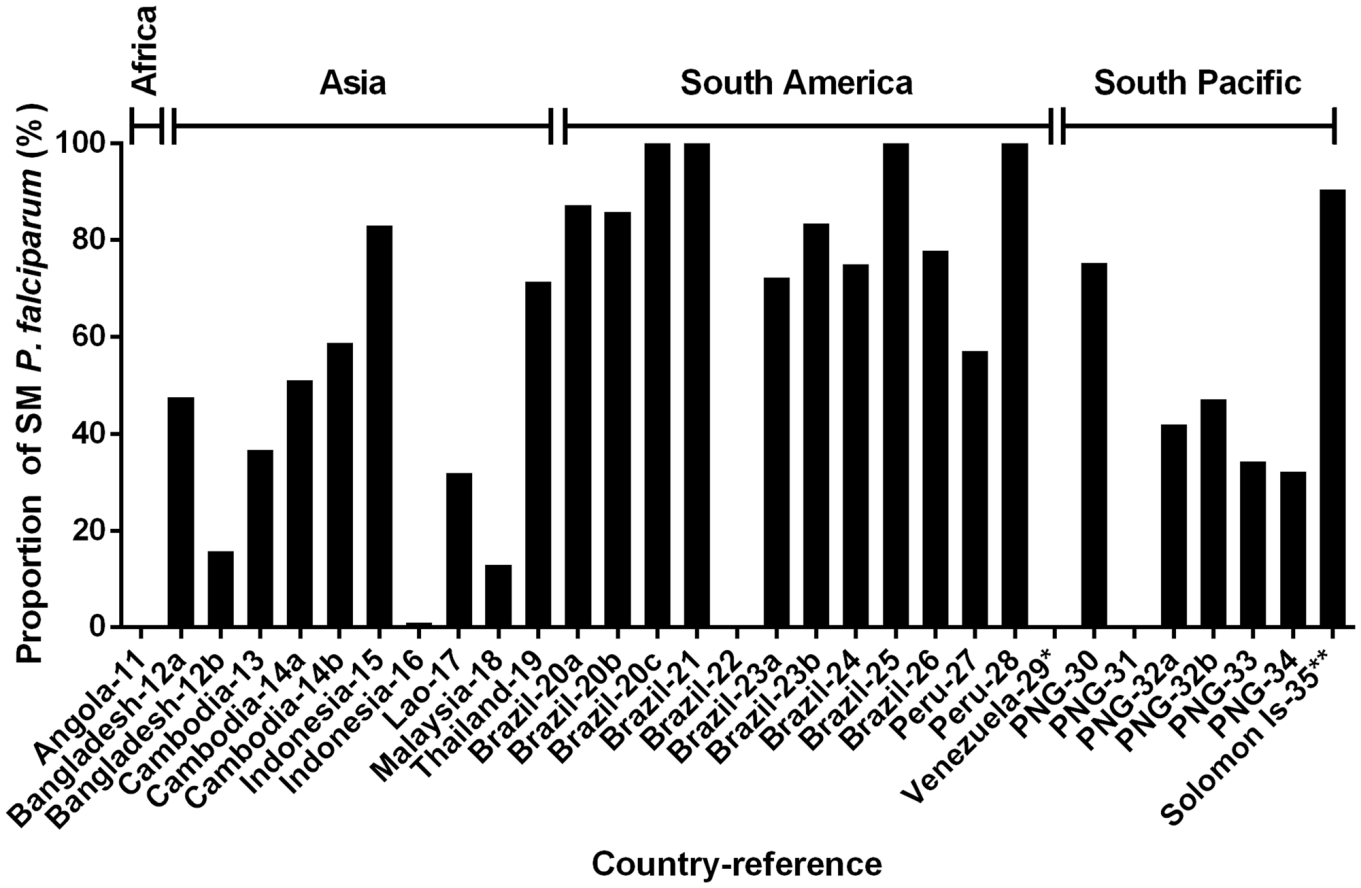
Relative proportion of sympatric SM (sub-microscopy) *P. falciparum* in cross-sectional surveys. Countries where data were collected and their corresponding references (detailed in Table 1) are shown on the x-axis.

The prevalence of SM *P. vivax* in the 31 community surveys analysed ranged from 0.2 to 48.6% (Fig. 1). The relative proportion of *P. vivax* not detected by LM (i.e. SM infections) ranged from 1.5% to 100.0%, with a mean of 69.5% (Fig. 3). The prevalence of SM *P. falciparum* ranged from 0.0% to 61.3%, constituting, on average, 55.7% of *P. falciparum* infections (range 0.0% to 100.0%, Fig. 4).

**Figure 5:**
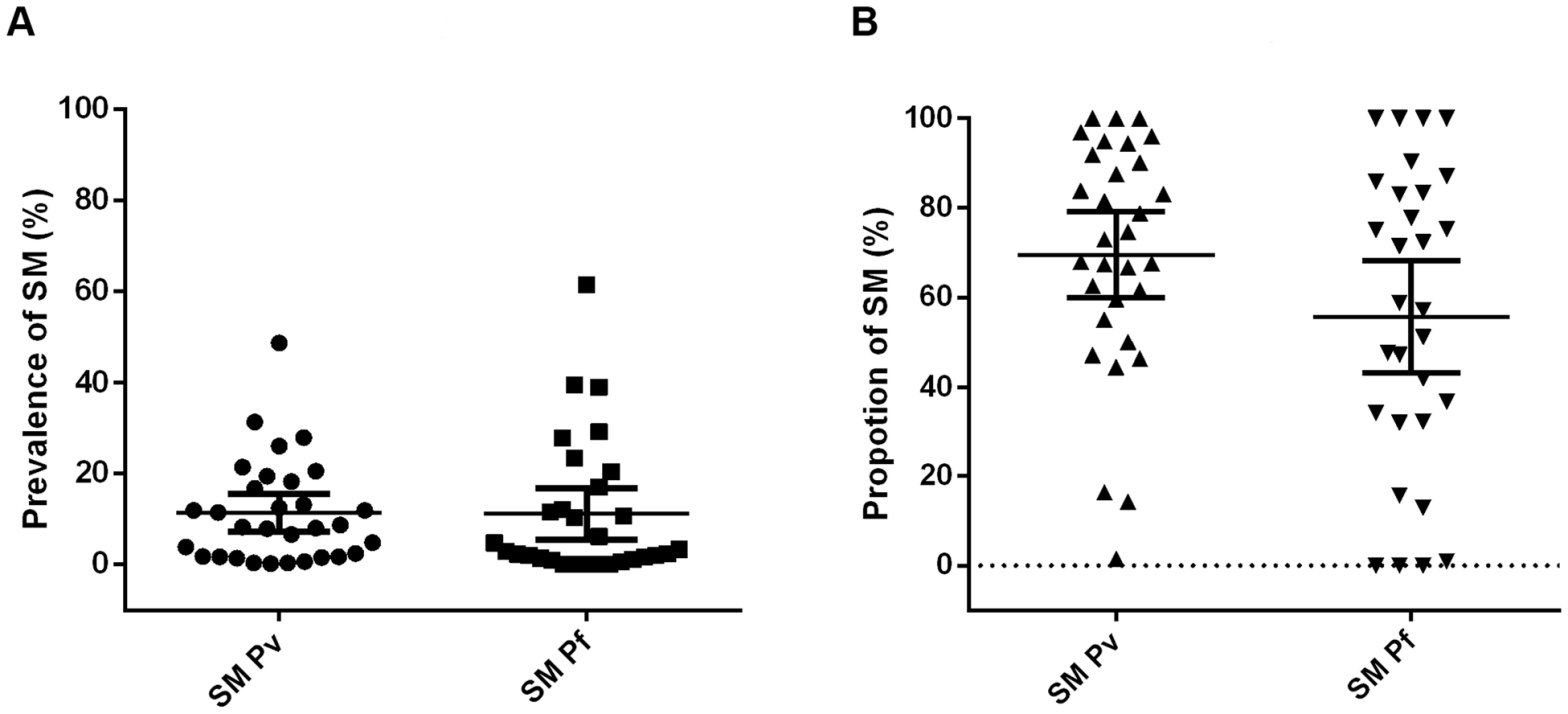
A) Comparison of SM (sub-microscopy) *P. vivax* and SM (sub-microscopy) *P. falciparum* prevalence (mean with 95% CI) in 31 cross-sectional surveys. B) Comparison of relative proportions of SM (sub-microscopy) *P. vivax* and *P. falciparum* (mean with 95% CI) in 31 cross-sectional surveys.

The prevalence of SM *P. vivax* in these communities was not significantly different to that of SM *P. falciparum* (Wilcoxon matched-pairs signed rank test, P = 0.78, Fig. 5A). However, the average relative proportion of SM was significantly higher in *P. vivax* infections compared to *P. falciparum* in the same study (Wilcoxon matched-pairs signed rank test, Median difference  = 5.2%, P = 0.045, Fig. 5B).

### Relationships between LM and PCR determined *P. vivax* prevalence

**Figure 6:**
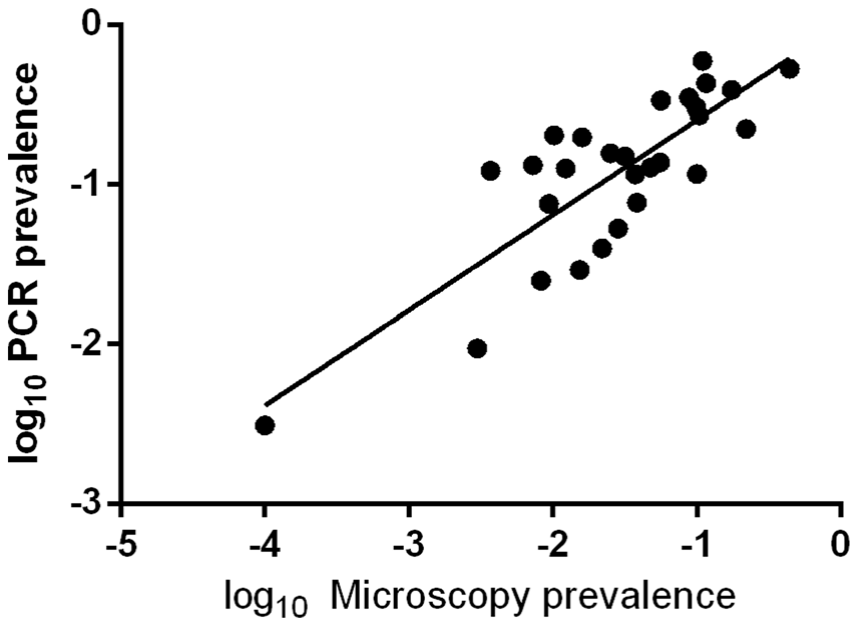
Relationship between LM (light microscopy) and PCR determined *P. vivax* prevalence in 31 cross-sectional surveys.

A positive relationship between LM and PCR determined *P. vivax* prevalence was observed (Fig. 6). LM *P. vivax* prevalence was a significant factor for predicting PCR *P. vivax* prevalence in community surveys (P<0.001, R^2^ = 0.675). The fitted regression equation is: log (PCR *P. vivax* prevalence)  = 0.596× log (LM *P. vivax* prevalence) – 0.003. Thus, PCR detectable *P. vivax* prevalence can be estimated based on LM *P. vivax* prevalence for community surveys using quality LM and PCR with similar sensitivities to those reported in the studies described here.

### Relationship between LM and relative proportion of SM *P. vivax*

**Figure 7:**
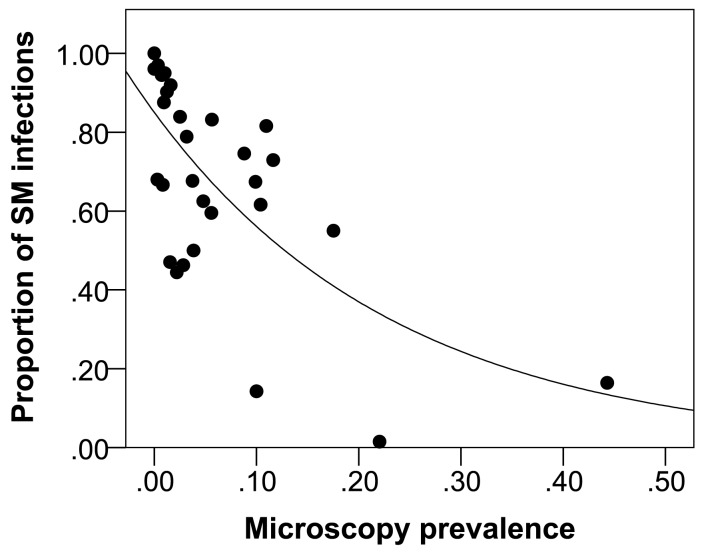
Relationship between LM (light microscopy) and SM (sub-microscopy) *P. vivax* prevalence in 31 cross-sectional surveys.

A negative relationship between LM *P. vivax* prevalence and proportion of SM *P. vivax* infections was identified (Fig. 7), with LM *P. vivax* prevalence identified as a significant factor for predicting the proportion of samples that are PCR positive/LM negative (SM) in cross-sectional surveys where the LM *P. vivax* prevalence is less than 45% (P<0.001, Deviance  = 7.32, df  = 29). The regression equation describing this relationship is: proportion SM *P. vivax*  =  exp (−0.162–4.163× LM *P. vivax* prevalence). The negative relationship observed remains when a furthest outlier was removed from the analysis.

### Pathogenesis of SM *P. vivax*

The pathogenesis of SM could be indicated by the percentage of infected individuals having malaria related symptoms at the time of the survey. Malaria symptoms include acute symptoms normally represented by fever, and chronic symptoms represented by anaemia. Data on the proportion of symptomatic *P. vivax* mono infections in PCR positive/LM negative subjects could only be extracted from six studies [16], [22], [25], [26], [27], [35], all of which were conducted in areas with relatively low transmission (LM *P. vivax* prevalence ranged 0.01% to 4.79%). The proportion of individuals with symptoms ranged from 0.0% to 11.4%, averaging 2.8%. This means that between 88.6 and 100.0% (average 97.1%) of individuals with SM *P. vivax* were asymptomatic. To ascertain whether these asymptomatic SM carriers will become symptomatic some time later, one study followed 25 LM negative/PCR positive subjects in South America for two months and found they remained asymptomatic over the 2 month duration [26].

The relationship between SM *P. vivax* and anaemia was investigated by Ladeia-Andrade et al. [23] in Brazil using two-level logistic regression models. After excluding LM positive subjects, and controlling for covariates such as age and sex, they concluded that the presence of SM *P. vivax* was a significant predictor of anaemia (OR = 1.92; 95% CI:1.14–3.23; P = 0.015).

### Factors contributing to the occurrence of SM infections

1. **Anti-parasite antibodies.** Antibodies against *P. vivax* are a marker of host immunity developed during previous infections. Four cross-sectional surveys [22], [25], [26], [28] included a serological investigation of anti-*P. vivax* antibodies using ELISA or IFA in parallel with LM and PCR. However, only two studies compared serological and PCR results. Versiani *et al* [26] observed that PCR positive/LM negative subjects were 2.45 fold more likely to have anti-PvMSP1 antibodies than PCR negative/LM negative subjects in Amazonas State of Brazil, and that the PCR positive/LM negative group had significantly higher titres of anti-PvMSP1 antibodies than the PCR negative/LM negative group [26]. Increased presence of anti-PvMSP1 antibodies was also noted for PCR positive/LM positive subjects (compared to subjects with no evidence of *P. vivax* infection), however there were too few cases positive by both PCR and LM (n = 15) to achieve statistical significance, or compare between SM and patent infections. A second study conducted in Bellavista, Peru observed that PCR positivity was significantly associated with the presence of anti-PvMSP1 antibodies [28], however no comparison was made between SM and patent infections.
2. **Age.** In many malaria endemic settings, the adolescent population has higher proportions of asymptomatic and low density Plasmodium infections than young children due to the development of clinical immunity after repeated exposure to *P. vivax* parasites. Therefore, age is a surrogate marker for acquired clinical immunity. Fifteen of the 25 publications (21 of the 31 surveys) described prevalence of *P. vivax* infection in different age groups, but only 12 publications (16 surveys) analysed the relationships between *P. vivax* LM or PCR prevalence and age. Seven publications (eight surveys) found no significant association between the relative proportion of SM *P. vivax* infection and host age [14], [15], [17], [22], [27], [28], [30], while five publications (eight surveys) reported that the relative proportion of SM *P. vivax* increased with age [12], [23], [31], [32], [34]. Neither the LM nor the PCR *P. vivax* prevalence was significantly different between the studies that did and did not find an association with age (Mann Whitney test, P>0.05).
3. **Microscopy QA and DNA extraction method for PCR.** The prevalence and relative proportion of SM *P. vivax* could vary because of differences in quality of microscopy and PCR. Only one [35] of the 25 publications (1 of the 31 surveys) stated the competency levels of microscopists according to the WHO accredited malaria microscopy competency assessment. Eleven publications (15 surveys) described the microscopists as experts, experienced, highly skilled or well-trained but did not provide information on qualifications. Fifteen of the 31 surveys reported some form of quality assurance (QA) of the microscopy including two independent microscopists reading and/or an expert referee to confirm positives, discordant slides and random selection of negatives, as well as PCR positive results. However, the presence or absence of a description for performing microscopy QA did not affect the average prevalence of SM (Mann Whitney test, P = 0.2447) or the average proportion of SM *P. vivax* (Mann Whitney test, P = 0.1626).
  While quality of microscopy largely depends on the competency of microscopists, quality of PCR can be particularly influenced by the type and volume of blood used to extract DNA. All surveys described the type of blood and DNA extraction method used for PCR analysis. Fourteen surveys used blood from filter paper while 17 surveys used whole blood (primarily>150 µL) for DNA extraction. The average LM prevalence of *P.* vivax was not significantly different between these two methods of DNA extraction (Mann Whitney test, P = 0.161). However, the average prevalence of SM *P. vivax* in community surveys determined by PCR using whole blood was significantly higher than that using blood from filter papers (Mann Whitney test, P = 0.004).
4. **Fever and drug use.** Antimalarial drug use could also affect parasite density at the time of survey and thereby increase the prevalence and relative proportion of SM *P. vivax*. Only one survey reported that recent antimalarial treatment (<4 weeks prior to survey) was associated with a significant increase in risk of having SM *P. vivax* infection [33].

### Prevalence of SM *P. vivax* infections in surveys among clinical malaria suspects and their proportions in different settings

**Figure 8:**
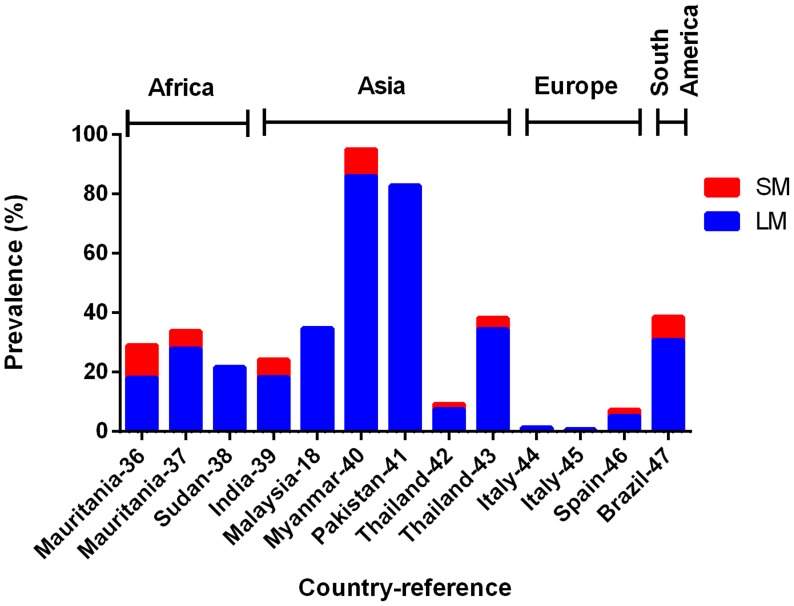
Prevalence of LM (light microscopy, blue bar) and SM (sub-microscopy, red bar) *P. vivax* in clinical malaria suspects. Total height of the bar (blue + red) represents the PCR prevalence.

**Figure 9:**
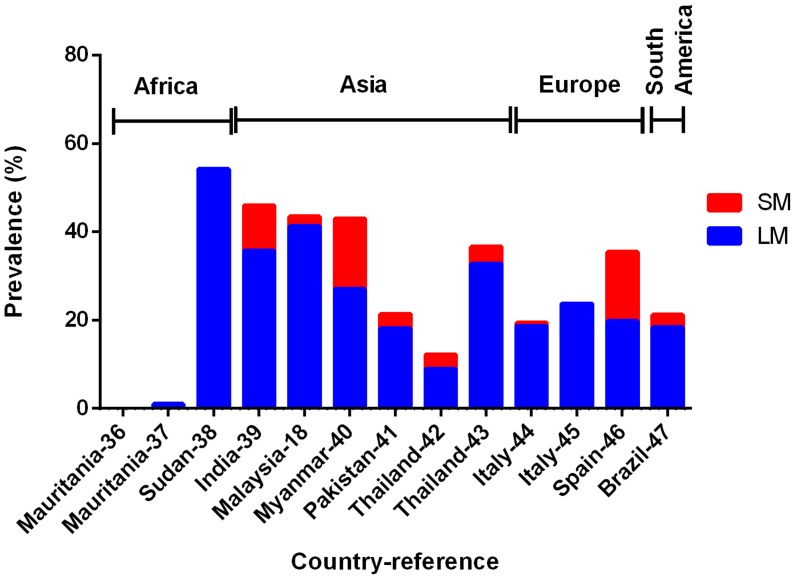
Prevalence of LM (light microscopy, blue bar) and SM (sub-microscopy, red bar) *P. falciparum* in clinical malaria suspects. Total height of bar (blue + red) represents PCR prevalence.

In 13 surveys of clinical malaria suspects conducted in different transmission settings (1997–2010), the prevalence of *P. vivax* infections determined by LM varied widely from 0.7% to 86.0% of the patients. Although PCR detected more *P. vivax* infections in some studies, overall it did not have a significantly higher prevalence than LM in fever patients (Wilcoxon matched-pairs signed rank test, P = 0.278, Fig. 8). In these same surveys, the LM *P. falciparum* prevalence rates ranged from 1.0% to 54.2% while PCR prevalence rates ranged from 1.0% to 46.9%. Similar to *P. vivax*, PCR did not detect a significantly higher number of *P. falciparum* infections than LM in these patients (Wilcoxon matched-pairs signed rank test, P = 0.123, Fig. 9). This suggests that amongst symptomatic patient populations SM *P. vivax* and *P. falciparum* are not prevalent.

## Discussion

Sub-microscopic malaria infections have been increasingly reported in malaria endemic areas, especially in places where malaria transmission intensities are relatively low and where malaria elimination is being targeted. In this paper, published articles from varying transmission intensities were reviewed and data extracted for secondary analysis with the purpose of providing an overview of SM *P. vivax* malaria in different settings including its prevalence and relative proportion of all detectable *P. vivax* infections, and relationship with LM prevalence, as well as consideration of the factors contributing to SM *P. vivax*.

The 31 cross-sectional surveys reviewed were conducted in areas where LM *P. vivax* prevalence ranged from 0 to 44%. Since entomological inoculation rate (EIR) was not reported in these surveys, we used LM *P. vivax* prevalence as an indicator of transmission intensity in each setting. One of the advantages of using LM prevalence instead of PCR prevalence is that LM prevalence has been used as a measure of transmission intensity in endemic countries for the past century. Our analysis showed that the PCR *P. vivax* prevalence was significantly higher than that LM *P. vivax* prevalence in these surveys, demonstrating the presence of a sub-population carrying SM *P. vivax* across all transmission settings. The prevalence of SM *P. vivax* infections ranged from 0.2% to 48.6%. SM *P. vivax* constitutes between 1.5% and 100% of all *P. vivax* infections detected by PCR (average 69.5%) in these settings, and this proportion is significantly higher than that of the sympatric *P. falciparum* infections. This suggests that without PCR or other comparably sensitive methods, an average of 69% of *P. vivax* infections would be undetected hence antimalarial treatment will usually not be sought. This results in a potentially large group of people carrying parasites that have the potential to transmit *P. vivax* [8], [9] and maintain the transmission cycle. Furthermore, the majority of these people are asymptomatic and may remain so for months [26].

Similar to the epidemiology of SM *P. falciparum* reported by Okell et al.[6], a negative relationship was identified between the relative proportion of SM *P. vivax* and LM *P. vivax* prevalence in 31 cross-sectional surveys. This suggests that a relatively larger proportion of *P. vivax* infections are SM in areas with low transmission intensity compared to areas with high transmission intensity, although SM *P. vivax* is prevalent in both type of settings. Hence, SM *P. vivax* poses more challenges to malaria control programs in areas where transmission intensities are already low and are progressing toward elimination.

Many factors can contribute to the occurrence and level of SM *P. vivax*. These include technical, parasite and host factors. From a technical point of view, the quality of microscopy is an important factor that can contribute to the level of LM *P. vivax*, and thus SM *P. vivax*. The prevalence and relative proportion of SM *P. vivax* would increase if the microscopist is relatively poor at detecting parasites, or decrease if there are false positive results (eg cell debris wrongly classified as a parasite). The correct speciation of parasites is also important in areas with both *P. falciparum* and *P. vivax*. This is a special concern since quality of field microscopy has been shown to be highly variable [48], [49], [50], [51], [52], [53], [54]. Interestingly, the description of microscopy QA was not found to be associated with the prevalence or relative proportion of SM *P. vivax* in the 31 cross-sectional surveys reviewed. This indicates that the quality of microscopy of these surveys was comparable between studies, irrespective of whether QA was reported or not.

Quality of PCR is also an important determinant for the level of SM *P. vivax*. It might be expected that a sensitive PCR would detect more SM *P. vivax* than a less sensitive PCR in a field survey. The PCR sensitivity is largely determined by the quality and amount of parasite DNA and by copy number of the target gene. Of the 31 cross-sectional surveys, 29 (93.5%) used a PCR-based assay targeting the parasite 18sRNA gene. Therefore, the sensitivity of these PCR methods would most likely be affected by the number of parasites added in each assay which is determined by the volume of blood used for DNA extraction and the concentration of parasite DNA. Seventeen of the 31 cross-sectional surveys used >150 µL of whole blood while 14 surveys used dried blood spot on filter paper which usually contain 5-20 µL of blood. As expected, the surveys using whole blood produced a significantly higher average prevalence of SM *P. vivax* compared to the surveys using filter paper blood. Although the final volume of DNA elusion could not be assessed, this difference is likely due to the higher number of parasites present in the larger volume of whole blood, resulting in more parasite DNA being added into each assay. This finding suggests that while filter paper helps to preserve blood in field conditions and assists with transportation of samples, the method is less sensitive than whole blood in detecting SM *P. vivax*. In order to maximize PCR sensitivity in detecting SM parasite infections, >150 µL of whole blood should be used when possible.

Parasite factors influencing SM *P. vivax* may include the growth characteristics and virulence of parasite strains, as well as genetic diversity, susceptibility to antimalarial drugs and relatedness of parasite strains. While the former, which is difficult to study in the field, may directly affect the parasite density, the latter could indirectly affect the parasite density through effective host immunity. For example, homogeneity of parasite population, i.e. lack of genetic diversity, is often reported in areas with low transmission intensity, such as Peru and South American countries [55], [56], [57]. This limited parasite genetic diversity could speed up the development of acquired immunity in a host population, which in turn could reduce parasite density in the host resulting in a higher proportion of SM infections. However, parasite diversity was not reported as part of any of the surveys reviewed. Recently, Gray et al [58] reported genetic diversity and parasite relatedness in Temotu, Solomon Islands as a follow up of the baseline survey [35]. In the baseline survey 75% of LM positive *P. vivax* infections had parasite densities below 100/µL with a further 44% of *P. vivax* infections being SM *P. vivax*; 94% of all *P. vivax* infections were asymptomatic at the baseline survey. However, genetic diversity of *P. vivax* population was very high and parasite haplotypes were not highly related [58]. This suggests that genetic diversity of *P. vivax* was not a major contributor to the high prevalence of SM *P. vivax* in this setting.

Host factors including non-specific immune responses, immunity status and human genetics could greatly impact parasite density, and hence the prevalence and proportion of SM *P. vivax*. Firstly, the non-specific immune response commonly associated with febrile illness has been hypothesized to directly reduce parasite density [59], [60] and any resultant antimalarial treatment can rapidly eliminate parasites. The effect of febrile illness and antimalarial treatment on SM *P. vivax* was investigated in one of the 32 cross-sectional surveys reviewed [33]. Lin et al. reported that fever episodes in the two weeks prior to sample collection, and antimalarial treatment 4 weeks prior, were associated with a significant reduction in risk of being detected by LM or PCR-based method [33]. Therefore, antimalarial treatment reduces both LM and SM *P. vivax* infections.

Host immune status could also be a major determinant of SM *P. vivax*. It has been reported that SM *P. falciparum* is more common in adults compared to children [6]. However, this pattern was not exactly repeated in *P. vivax*. Of the 16 surveys that analysed the relationship of SM *P. vivax* with age, eight reported the relative proportion of SM *P. vivax* was not associated with age, while the other eight reported that the relative proportion of SM *P. vivax* increased with age. This difference may be related to the transmission intensities in study regions, however a comparison of the average LM *P. vivax* prevalence between these two groups did not show a significant difference. One other possibility may be difference in geographical or host population; the group of surveys reporting no association with age were mostly conducted in Southeast Asia and South America, while those reporting an association included four surveys conducted in PNG. The presence and quantity of anti-PvMSP1 antibodies have also been associated with SM *P. vivax* in two surveys conducted in South America [26] [28]. This suggests that serology may help detect SM *P. vivax* carriers.

Associations between other host factors such as polymorphisms in Duffy antigen, haemoglobin or G6PD and SM *P. vivax* infections were not investigated in any of the 31 cross-sectional surveys reviewed. Further studies are required to ascertain the contribution of host genetics to SM *P. vivax* infections.

Based on findings of six surveys, between 89% and 100% (average 97.5%) of SM *P. vivax* infected subjects were asymptomatic at the time of survey and it was not well documented whether these people progressed to develop LM detectable parasitemia at a later time. One study followed 25 LM-/PCR+ subjects and found them all asymptomatic after a 2 month follow up [26]. If these individuals remain asymptomatic then they would not be identified by either active or passive case detection based on LM or RDTs with a similar sensitivity to LM.

Any individual infected with blood stage *P. vivax*, regardless of the level of parasitemia, is likely carrying dormant hyponozoites in the liver and is thus likely to relapse weeks to months after the primary infection. Future relapses in these individuals can only be limited by treatment that includes antimalarials (specifically, primaquine) targeting the *P. vivax* hypnozoites. Therefore, to prevent relapse WHO recommends that in low transmission areas patients with *P. vivax* infection, who are not G6PD deficient, receive treatment against both blood (such as chloroquine) and hypnozoite (primaquine, 0.25 or 0.5mg/kg/day once a day for 14 days) stages [61]. However, SM *P. vivax* infections are not detectable using LM or RDTs, as such these infected individuals would not receive any anti-malarial therapy for *P. vivax* blood or liver stages and thus continue to transmit *P. vivax*. The prevalence of this undetectable and untreated SM *P. vivax* population creates a major challenge to malaria control and elimination programs. Strategies for detecting and treating infected cases (patent and symptomatic infections) alone will unlikely interrupt transmission because SM *P. vivax* infections and their future relapses will continue to feed into transmission. Comparing to SM *P. falciparum*, SM *P. vivax* infections represent a more important transmission reservoir due to multiple relapses occurring over a long period of time. To accelerate malaria elimination i.e. the complete interruption of malaria transmission and total removal of the disease burden of malaria such as anaemia, some form of mass screening using PCR-based or comparably sensitive methods and radical cure approach would be required to identify and treat all SM *P. vivax* infected subjects. This strategy would be expected to have more impact on *P. vivax* transmission than on *P. falciparum* transmission because appropriate management of SM *P. vivax* will not only stop transmission from current SM infections, but also prevent future relapses and transmissions resulting from these relapses. However, because the conventional PCR based assays are expensive to implement and difficult to perform under field conditions, mass screening will not be practical before more sensitive and specific field deployable diagnostic tests become available. Cost-effectiveness studies will be needed to properly evaluate the options available for *P. vivax* elimination strategies for different transmission settings. In the interim, the PCR prevalence of *P. vivax* could be estimated based on the positive relationship between PCR *P. vivax* prevalence and LM *P. vivax* prevalence identified in this study. If the prevalence of PCR positive *P. vivax* is much higher than that of LM, mass drug administration may be an option for elimination but it also has its operational challenges, risks and potential limitations [62].

In contrast to cross-sectional surveys, SM *P. vivax* was much less prevalent in clinical malaria suspects. Overall, the PCR prevalence for *P. vivax* or *P. falciparum* was not significantly higher than that of LM in 13 surveys of febrile patients. This could be due to relatively high parasite density in symptomatic patients. This re-emphasizes that quality LM and RDTs are adequate tools for case management of both *P. vivax* and *P. falciparum* patients.

In summary, SM *P. vivax* is prevalent across different geographic areas with varying transmission intensities constituting, on average, 69.5% of all *P. vivax* infections. The relative proportion of SM *P. vivax* is significantly higher than that of the sympatric *P. falciparum* in these settings and is higher in areas with relatively low transmission intensity. These SM *P. vivax* infections not only have negative health impact on the infected individual, but will also contribute to *P. vivax* transmission both from the current infection and subsequent relapses, and thus present a major challenge for malaria elimination programs. This review seeks to provide malaria control and elimination programs with estimates of the prevalence and proportion of SM *P. vivax* infections in their settings, and to highlight the importance of developing diagnostic tools for detecting SM *P. vivax* infections in order to support elimination strategies. Strategies for tackling both patent and SM *P. vivax* are critical for eliminating *P. vivax*.

## Supporting Information

S1 Checklist **PRISMA checklist.**
(DOC)

S1 Diagram **PRISMA flow diagram.**
(DOCX)
