# Supplementary material for: Systematic Review of Sub-microscopic P. vivax Infections: Prevalence and Determining Factors
Source: PLoS Negl Trop Dis. 2015 Jan 8;9(1):e3413. doi: 10.1371/journal.pntd.0003413 (PMC4288718; doi:10.1371/journal.pntd.0003413)
Supplement: S1 Diagram — PRISMA flow diagram. (DOCX) [file pntd.0003413.s002.docx]

**PRISMA 2009 Flow Diagram**


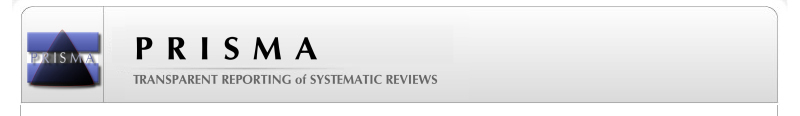


Studies included in quantitative synthesis (meta-analysis)
(n =38 )

Studies included in qualitative synthesis
(n =38 )

Full-text articles excluded, with reasons
(n = 98 )

Full-text articles assessed for eligibility
(n = 136 )

Records excluded
(n = 3 )

Records screened
(n = 139 )

Records after duplicates removed
(n = 0 )

Additional records identified through other sources
(n = 0 )

## Identification

## Eligibility

## Included

## Screening

Records identified through database searching
(n = 139 )
